# Evolution of mating behavior between two populations adapting to common environmental conditions

**DOI:** 10.1002/ece3.1454

**Published:** 2015-03-18

**Authors:** Margarida Bárbaro, Mário S Mira, Inês Fragata, Pedro Simões, Margarida Lima, Miguel Lopes-Cunha, Bárbara Kellen, Josiane Santos, Susana A M Varela, Margarida Matos, Sara Magalhães

**Affiliations:** cE3c - Centre for Ecology, Evolution and Environmental Changes, Faculdade de Ciências, Universidade de LisboaCampo Grande, 1749-016, Lisboa, Portugal

**Keywords:** *Drosophila subobscura*, experimental evolution, laboratory adaptation, latitudinal cline, mating behavior, reproductive barriers

## Abstract

Populations from the same species may be differentiated across contrasting environments, potentially affecting reproductive isolation among them. When such populations meet in a novel common environment, this isolation may be modified by biotic or abiotic factors. Curiously, the latter have been overlooked. We filled this gap by performing experimental evolution of three replicates of two populations of *Drosophila subobscura* adapting to a common laboratorial environment, and simulated encounters at three time points during this process. Previous studies showed that these populations were highly differentiated for several life-history traits and chromosomal inversions. First, we show initial differentiation for some mating traits, such as assortative mating and male mating rate, but not others (e.g., female mating latency). Mating frequency increased during experimental evolution in both sets of populations. The assortative mating found in one population remained constant throughout the adaptation process, while disassortative mating of the other population diminished across generations. Additionally, differences in male mating rate were sustained across generations. This study shows that mating behavior evolves rapidly in response to adaptation to a common abiotic environment, although with a complex pattern that does not correspond to the quick convergence seen for life-history traits.

## Introduction

Populations from different environments are likely to become genetically and phenotypically differentiated, either due to local adaptation to ecologically distinct environments (Kawecki and Ebert 2004) or to the accumulation of genetic incompatibilities (Corbett-Detig et al. 2013). This differentiation can result in the evolution of reproductive isolation, potentially leading to speciation (Schluter 2009).

Such differentiated populations may subsequently migrate into the same location. This co-occurrence in a common environment may affect the degree of reproductive isolation between these populations. Studies addressing this issue traditionally focus on the consequences of interpopulational encounters for reproductive isolation. Indeed, co-occurring populations are expected to compete for resources, potentially leading to character displacement (Rundle and Nosil 2005), thereby enhancing reproductive isolation. Moreover, encounters may result in interpopulation matings, leading either to reproductive character displacement (including reinforcement, Pfennig and Pfennig 2009) or to higher gene flow among populations (reviewed in Fry 2009). Which of these possibilities holds true probably depends on the degree of population differentiation before migrating to a common environment.

Apart from such biotic interactions, natural selection resulting from abiotic factors may also affect the evolution of reproductive isolation when allopatric populations converge ecologically. For example, adaptation to the abiotic environment may result in individuals being better at acquiring mates, and this may affect the relative success of such mates, as compared to those from other populations. The question is, then, how does mating behavior evolve during adaptation to the same abiotic conditions? Does reproductive isolation wane because populations are converging for several traits, or is there a reinforcement of reproductive barriers? Curiously, to date, no study has addressed the role of adaptation to a common novel abiotic environment on reproductive isolation.

Experimental evolution studies have multiplied in recent years, with the increasing notion of the power of this methodology (Kawecki et al. 2012b; Magalhães and Matos 2012). Such studies focus mostly on the evolution of life-history traits (reviewed in Kawecki et al. 2012a). However, recently, several studies have addressed the evolution of behavioral traits associated with mating. Such is the case of studies that manipulated the opportunity for sexual selection (e.g., monogamy vs. polyandry/polygamy or the operational sex ratio), then measured how this affected several mating traits in males and/or females of several organisms, such as *Drosophila* (Crudgington et al. 2005; Rundle et al. 2005; Snook et al. 2005; Bacigalupe et al. 2008; Debelle et al. 2014), dung flies (Hosken and Ward 2001; Hosken et al. 2001), or beetles (Simmons and García-González 2008; Fricke et al. 2010; Gay et al. 2011; Michalczyk et al. 2011); see also a review in Edward et al. (2010). Some studies analyzed how sexual conflict affected the evolution of reproductive isolation (Martin and Hosken 2003; Wigby and Chapman 2006; Bacigalupe et al. 2007). In all these studies, it was expected that mating traits would be modified by male–female encounters. In another set of experimental evolution studies, populations were exposed to different habitats (i.e., populations were subjected to divergent selection) and tests were performed to assess how this affected the evolution of reproductive isolation (reviewed in Fry 2009). However, the opposite, which is how experimental evolution of differentiated populations adapting to a novel common environment affects mating traits and reproductive isolation among them, has not been tackled. This may shed light on the evolutionary trajectories of such traits under ecological convergence.

*Drosophila subobscura* exhibits latitudinal clines for chromosomal inversions and body size, resulting in high differentiation among populations from the cline extremes (Gilchrist et al. 2004; Rezende et al. 2010). The evolutionary dynamics of life-history traits of *D. subobscura* populations introduced to a novel, similar, environment (the laboratory) is well documented (Matos et al. 2002; Simões et al. 2008). Recently, we showed that populations derived from contrasting latitudes in Europe converged for several phenotypic traits within a few generations (Fragata et al. 2014a). These populations presented initial high differentiation both in life-history traits and inversion frequencies (Fragata et al. 2014a,b). The degree of early phenotypic differentiation among such populations was much higher than that shown by populations derived from neighboring locations (Fragata et al. 2014a). This suggests that history rather than sampling effects alone affect the differentiation among populations from different latitudes within the cline. Still, how mating preferences and reproductive barriers evolve in these populations is unknown. Here, we address this issue using the two populations from the extremes of the cline. We exclude encounters among individuals from differentiated populations during the evolutionary process. By doing so, we propose a scenario where two migrating populations first adapt independently to the novel common environment and only meet after a certain number of generations. Once they do, the question is whether there will be evidence of reproductive isolation in their mating behaviors or not. Specifically, we ask (a) Are populations from different ecological environments initially differentiated for mating behavior traits? (b) If so, how do these traits evolve during adaptation to a novel, common environment? How does this adaptation process, which occurs independently in populations from different foundations, affect reproductive isolation? Answering these questions will complement our knowledge of adaptive evolution and shed light on the role of abiotic factors in the evolution of reproductive barriers among populations.

## Materials and Methods

### Foundation and maintenance of populations

*Drosophila subobscura* individuals were collected in August 2010 from two locations: Adraga (Ad), Portugal, and Groningen (Gro), Netherlands. Females from the first two generations were maintained in separate vials, to equalize their contribution to the next generation. Inbreeding was avoided by crossing females with males from different vials. At the third generation, an equal number of offspring of each female were randomly mixed, giving rise to the outbred populations (see details in Fragata et al. 2014a). At generation four, these foundations were threefold replicated (Populations Ad_1–3_ and Gro_1–3_) and maintained in large numbers under standard laboratory conditions (Simões et al. 2008; Fragata et al. 2014a).

### Mating behavior assays

No-choice and female-choice experiments were performed at generations 5, 10, and 17. Virgins were sexed and kept in vials during 10 days before the experiments.

The design used to define the crosses was as follows: mating pairs were with females and males either from the same population (e.g., Ad_1_ males × Ad_1_ females) or from different foundations (e.g., Ad_1_ females × Gro_1_ males), but not between replicate populations from the same foundation (e.g., Ad_1_ females × Ad_2_ males); for crosses between foundations, we assigned arbitrarily same numbers to define which populations were involved (e.g., Ad_1_ females × Gro_1_ males). Thus, our design is a block design involving three random blocks, orthogonal to the fixed effects to be tested (see below) allowing thus to test for several interactions of interest. Specifically, block 1 includes matings involving Ad_1_ and Gro_1_ individuals (homogamic: Ad_1_ × Ad_1_ and Gro_1_ × Gro_1_, and heterogamic Ad_1_ × Gro_1_, being either male × female or female × male), and the same logic for blocks 2 and 3. This contrasts with other designs that are used in mating experiments (e.g., Bacigalupe et al. 2007) where replicate populations are nested within cross-types.

In no-choice experiments, a homogamic (Gro × Gro or Ad × Ad) or heterogamic (Gro × Ad or Ad × Gro) pair was placed in each vial. Experiments consisted of three blocks (each with one population from each foundation), with approximately 25 series of eight mating pairs, two of each type. Pairs were observed during 90 minutes or until a mating occurred. When a mating event occurred, two parameters were measured (in seconds): mating latency (elapsed time to the beginning of copulation), and mating duration (time spent copulating).

In female-choice experiments, two males, each from a different foundation, were placed with one female (Ad or Gro). Two days before the assays, males were randomly marked with an innocuous powder, green or red. Other set-up details were as no-choice experiments, but with 4-vial series. As male size may correlate with female mate choice (Monclus and Prevosti 1971), this trait was estimated by measuring wings as in previous studies (Fragata et al. 2010).

Mating frequency was registered in both no-choice and female-choice experiments.

### Statistical analysis

All statistical analyses were performed using Microsoft Excel, Statsoft Statistica and R version 2.11.1 (R Foundation for Statistical Computing, Vienna, Austria). General linear mixed models (GLMMs) were analyzed using the lme4 package (Bates et al. 2014).

#### No-choice experiments

In the analyses to test for initial differentiation (generation 5), only data from homogamic crosses were used, whereas in the analyses to test for evolutionary changes across generations data from both homogamic and heterogamic crosses were used.

The frequency of mated and not-mated individuals was tested among and within generations using GLMMs with a binomial distribution. We used a backward stepwise procedure for model selection. We tested for initial differentiation between foundations with block (with three categories 1, 2, 3) as random factor and foundation (with two categories Ad and Gro) as fixed factor, including significant interaction terms. Evolutionary changes among generations were analyzed with block as random factor, female population (i.e., the source population for the female in the cross, with two categories Ad and Gro) and male population (with two categories Ad and Gro) as fixed factors and generation (with three categories 5, 10, 17) as covariate. This analysis was complemented with a per-generation analysis with the same factors, but without the covariate generation.

The remaining no-choice experiments data (mating latency and mating duration) were analyzed with ANOVAs, with random factor block being excluded if this factor and its respective interactions were not significant. We tested initial differentiation between foundations, with foundation (*Fd*) as fixed factor and block (*B*) as random factor, including their interaction (model 1). We then tested for the occurrence of evolutionary changes with male population (*M*) and female population (*F*) as fixed factors, and generation (*G*) as a covariate, including their interactions (model 2). This analysis was complemented by per-generation ANOVAs with the same factors as the previous analysis, but without the covariate generation (model 3).

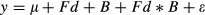
1

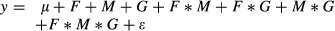
2

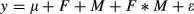
3

#### Female-choice experiments

The initial frequency (generation 5) of assortative mating (homogamic versus heterogamic matings) was tested for each foundation with GLMMs with a binomial distribution. We used a backward stepwise procedure for model selection. The initial model comprised block and vial as random factors, male population as fixed factor and male size as a covariate. Analysis of evolutionary changes among generations was performed with data from both foundations, with block and vial as random factors, male population and female population as fixed factors and male size and generation as covariates. All possible interaction terms were defined in the initial models. This was complemented by a per-generation analysis, without the covariate generation.

To measure reproductive isolation between foundations, an isolation index (*II*) was calculated for each population at each generation assayed using the formula (Dodd 1989):

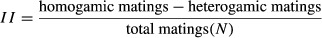


*II* ranges from −1 to +1; *II *= 0 indicates random matings; *II* > 0 assortative mating and *II* < 0 disassortative mating. Differences in *II* between foundations were analyzed using Mann–Whitney *U*-tests. Changes in *II* across generations were assessed using ANOVAs both within foundations (with block as random factor and generation as covariate – model 4) and between foundations (with foundation as fixed factor, block as random factor, and generation as covariate – model 5). Nonsignificant random factors or interactions were excluded from the model.

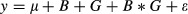
4

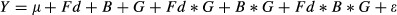
5

Size differences among males in each female-choice experiments-assayed generation were tested using ANOVAs, with block as random factor and male population as fixed factor, including their interaction (model 6). To test for differences across generations, the covariate generation and its respective interaction were added to the previous model (model 7). This trait was then added as a covariate to the GLMM analyses, to test whether it affected the significance of traits.

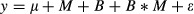
6

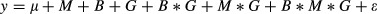
7

## Results

In all analyses, the block effects and their interaction with the factors under study were not significant, so they were dropped from all analyses.

In no-choice experiments, at generation 5, Gro individuals mated significantly more often than Ad individuals (*Z* = 2.930, *P *=* *0.034). Gro females mated significantly more often than Ad females at generation 5 (*Z* = 2.385, *P *=* *0.0171). This difference became nonsignificant at generation 10 (Table S1, *Z* = 1.5531, *P *=* *0.1204) and marginally significant at generation 17 (*Z* = 1.901, *P *=* *0.0574). Although Gro males always mated more often than Ad males, the factor male population was only marginally significant at generations 5 (*Z* = 1.710, *P *=* *0.0874), 10 (*Z* = 1.745, *P *=* *0.0809), and 17 (*Z* = 1.901, *P *=* *0.0574). The mating frequency increased significantly among generations across foundations (Fig. S1, Table S1, *Z* = 12.045, *P *<* *0.0001) with Ad individuals mating significantly less often than Gro individuals (male population: *Z* = 3.045, *P *=* *0.023; female population: *Z* = 3.276, *P *=* *0.0011), and no significant interactions between generation and either male population or female population.

**Figure 1:**
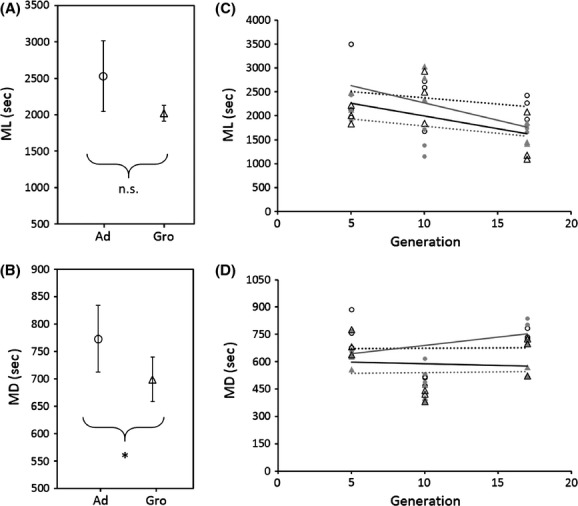
(A, B) – Means of (A) mating latency (ML) and (B) mating duration (MD) for homogamic crosses in no-choice experiments at generations 5. Ad (circles); Gro (triangles). Error bars correspond to standard errors. *P <* *0.05; n.s. nonsignificant. (C, D) – Evolutionary trajectories for (C) ML and (D) MD in no-choice experiments for Ad × Ad (dashed black line, circles), Ad ♀ × Gro ♂ (dashed gray line, full circles) Gro × Gro (black line, triangles), Gro ♀ × Ad ♂ (gray line, full triangles). Data points show mean values for each block.

At generation 5, differences in mating latency among homogamic crosses were not significant (Fig.1A, *F*_2,51_ = 0.7198, *P *=* *0.4826). However, Ad couples spent significantly more time mating than Gro couples (Fig.1B, *F*_2,51_ = 7.7109, *P *=* *0.0269). Mating latency decreased and mating duration increased significantly across generations in both foundations (Fig.1C and D, Table1, effect of generation). Namely, the mating latency of Gro females decreased across generations, whereas that of Ad females showed only a slight decline leading to a significant female * generation interaction (Table1, Fig.1). This led to an increase of differences between foundations throughout the generations, with mating latency becoming significant from generation 10 onwards. Differences in this trait also increased across generations in males, with Ad becoming significantly higher than Gro from generation 10 onwards (Table1). Ad males spent significantly more time mating than Gro males in all generations. Additionally, the interaction generation * male population was marginally significant for mating duration, suggesting different evolutionary dynamics in this trait between Ad and Gro males.

**Table 1:** Two-way ANOVA for mating latency (ML) and mating duration (MD) at generations 5, 10, and 17 followed by an ANCOVA for ML and MD across generations in the no-choice experiments. Statistically significant values (*P *<* *0.05) are marked in bold

Source	ML				MD			
	df	MS	*F*	*P*	df	MS	*F*	*P*
Generation 5								
Female population	1	39 369	0.1965	0.6584	1	4947	0.0676	0.7954
Male population	1	66 660	3.3268	0.0709	1	30 255	4.1314	**0.0445**
Female population ^*^ Male population	1	78 935	0.0394	0.8430	1	21 540	2.9414	0.0892
Error	111	20 037			110	73 232		
Generation 10								
Female population	1	21 425	10.4211	**0.0015**	1	28 335	0.847	0.3587
Male population	1	13 914	6.7678	**0.0101**	1	55 520	16.598	**<0.0001**
Female population ^*^ Male population	1	37 467	1.8224	0.1788	1	11 867	0.355	0.5522
Error	171	20 559			170	33 451		
Generation 17								
Female population	1	21 605	10.7649	**0.0012**	1	21 394	3.029	0.1540
Male population	1	14 447	7.1982	**0.0077**	1	26 185	37.071	**<0.0001**
Female population ^*^ Male population	1	28 787	0.1434	0.7051	1	20 063	0.284	0.6455
Error	313	20 070			311	70 637		
Across generations								
Generation	1	36 950	17.8013	**<0.0001**	1	64 8637	9.2880	**0.0024**
Female population	1	98 325	4.7369	**0.0299**	1	11 778	0.1687	0.6815
Male population	1	66 875	3.2218	0.0732	1	28 199	0.4038	0.5253
Generation ^*^ Female population	1	14 451	6.9622	**0.0085**	1	5264	0.0754	0.7838
Generation ^*^ Male population	1	32 115	0.1547	0.6942	1	24 127	3.4548	0.0636
Female population ^*^ Male population	1	82 254	0.3963	0.5293	1	11 343	1.6243	0.2030
Generation ^*^ Female Population ^*^ Male population	2	15 311	0.0738	0.7860	2	11 899	1.7039	0.1923
Error	599	20 757			595	69 836		

In female-choice experiments, at generation 5, females from both foundations mated significantly more often with Gro than with Ad males, although this result was only marginally significant for Ad females (*Z* = 1.870, *P *=* *0.0615). The frequency of assortative mating was, therefore, higher for Gro than for Ad females. Also, Gro males mated significantly more often than Ad males in each generation (generation 5: *Z* = 4.262, *P *<* *0.0001; generation 10: *Z* = 2.197, *P *=* *0.028; generation 17: *Z* = 3.272, *P *=* *0.0011) and across generations (Table2, *Z* = 5.665, *P *<* *0.0001) with no significant generation * male population interaction. Also, despite the fact that the best model included male size, indicating that this trait plays a role in the mating outcome, the covariate male size was never significant (Table2).

**Table 2:** Results of binomial tests for the number of mated and not-mated males in female-choice experiments at each assayed generation and across generations. Statistically significant values (*P *<* *0.05) are marked in bold

Generation	Source	*Z*	*P*
5	Male population	4.262	**<0.0001**
	Male size	−0.678	0.498
10	Male population	2.197	**0.028**
	Male size	−1.250	0.211
17	Male population	3.272	**0.001**
	Male size	−0.207	0.836
Across Generations	Male population	5.665	**<0.0001**
	Male size	−0.626	0.531
	Generation	−0.080	0.936

**Figure 2:**
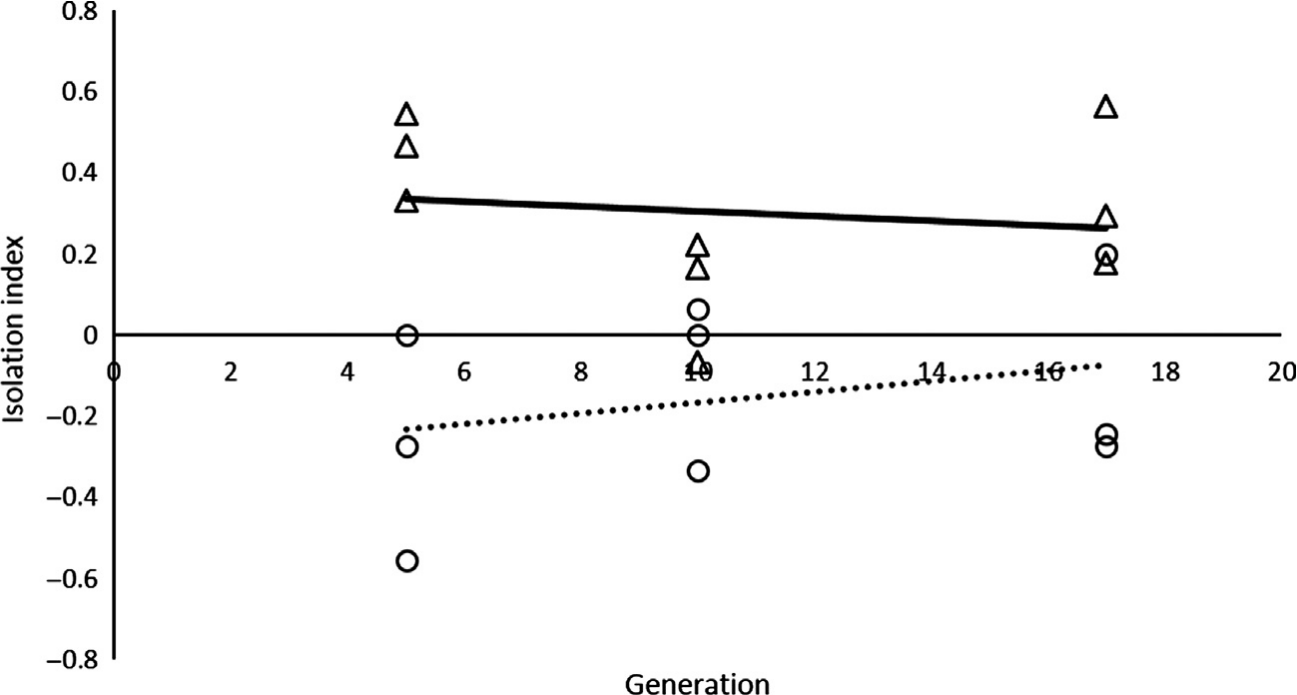
Isolation Index (II) at generations 5, 10, and 17. Dashed line, circles, Ad females; full line, triangles, Gro females.

The *II* was significantly different between foundations at generation 5 (*Z* = −1.96396, *P *=* *0.0495) and across generations (Table S2, factor foundation: *F* = 6.5372, *P *=* *0.0228). Nevertheless, the negative *II* of Ad females approached zero across generations (Fig.2). Finally, Gro males were significantly bigger than Ad males in all generations (generation 5: *Z* = 478.256, *P *=* *0.0019; generation 10: *Z* = 25.47, *P *=* *0.0355; generation 17: *Z* = 50, *P *=* *0.0169) and across generations (*Z* = 239.928, *P *=* *0.0039).

## Discussion

In this study, we examined the role of a novel, common environment in the evolution of reproductive isolation between two sets of laboratory populations of *D. subobscura*, derived from the extremes of the species European cline. Populations were maintained separately but under the same laboratorial conditions, to single out the role of abiotic factors (i.e., the laboratory environment) in behavioral traits associated to mating. The evolutionary trajectories of three behavioral traits – mating latency, mating duration, and mate preference – were analyzed. We observed that *D. subobscura* individuals from both foundations differed in mating duration and in the degree of assortative mating. These traits evolved during adaptation to the novel, common environment.

Initially, Adraga males mated for longer time periods than Groningen males. A longer mating duration may improve male reproductive success by increasing sperm transfer (e.g., Simmons et al. 1999), although the evidence for this in *Drosophila* is controversial (Bretman et al. 2009; Lupold et al. 2010). Also, *D. subobscura* is monandrous, and hence, adaptation to sperm competition is unlikely (but see Lizé et al. 2011). Still, mating duration increased in all populations across generations, suggesting an adaptive value for longer mating durations. Moreover, mating latency decreased across generations, particularly in Groningen females, leading to divergence between foundations for this trait. Possibly, there is a direct advantage of shorter latency, in response to higher male–male competition in the laboratory environment (e.g., Michalczyk et al. 2011; but see Bacigalupe et al. 2008). Another hypothesis is that such traits evolved as a by-product of selection for other characters (for example, selection for early reproduction observed in these populations Fragata et al. 2014a). In choice experiments, Groningen males obtained a higher proportion of matings with both types of females. Groningen males may be fitter, particularly when competing with Adraga males, for example, due to their bigger size, as this trait may affect mating speed in *D. subobscura* (Monclus and Prevosti 1971). In that case, females would choose adaptively (or males win the competition). Alternatively, mate choice may be the product of sensory biases (Ryan and Rand 1993). In any case, across generations, assortative mating was maintained in Groningen populations, while disassortative mating decreased in Adraga populations. This suggests that Adraga males become fitter due to laboratory adaptation, hence gaining more matings when competing with Groningen males at later generations.

Previous studies using these populations revealed evolutionary convergence of several phenotypic traits following laboratory adaptation (Fragata et al. 2014a). Here, we do not find evidence for convergence in behavioral mating traits. Indeed, we found that differences among some traits were maintained across generations (i.e., the traits evolved in parallel in populations from both foundations), such as mating duration and male mating frequency, whereas others diverged, such as mating latency. For the isolation index, although the statistical analysis of the evolutionary trajectories was not significant, Groningen individuals maintained their assortative mating, whereas Adraga individuals mated significantly more with individuals from Groningen initially, and evolved toward random mating. Thus, it seems that this trait also follows foundation-specific evolutionary dynamics, which are not compatible with evolutionary convergence. Possibly, the evolution of interacting phenotypes (Moore et al. 1997) follows more idiosyncratic trajectories than other traits, as suggested by theoretical studies (Agrawal et al. 2001). Interestingly, these populations also did not show convergence at the inversion frequency level (Fragata et al. 2014b). Perhaps the lack of convergence observed for mating behavior relates with effects of inversions that maintained differences between populations.

When populations from different localities arrive to a common environment, their individuals are expected to be differentiated due to local adaptation in each ancestral environment. Supposing that populations meet shortly after arriving into the novel environment, this may result in individuals minimizing contact with the other population, thereby reinforcing their isolation. In contrast, if these populations reside for long enough in the common environment but without having physical contact, they may converge for several traits, possibly facilitating gene flow once they meet. While the quick and clear pattern of convergent evolution presented by our populations for life-history traits points in this direction (Fragata et al. 2014a), here, we saw that a similar facilitation of gene flow is not occurring for mating behavior traits. Indeed, Adraga females were initially keen to mate with males from Groningen, and this preference disappeared after some generations of laboratory adaptation. This suggests that random mating increases as populations adapt to a common environment, leading to a stronger reproductive isolation, as Adraga females reduce their preference for males from the other population. Therefore, counter-intuitively, gene flow among these populations is more likely to occur before adaptation to a common environment. Again, this may stem from the idiosyncratic nature of evolutionary trajectories for behavioral traits (Moore et al. 1997). Whether our results illustrate a general feature remains to be established.

In summary, we show that mating behavior and reproductive isolation are labile traits with rapid evolution in response to abiotic conditions. Therefore, adding a temporal component to reproductive isolation studies will help addressing how reproductive barriers affect the outcome of secondary contacts.

## Supporting Information

**Figure S1.** Evolutionary trajectories for percentage of matings in no-choice experiments for Ad × Ad (dashed black line, circles), Ad ♀ × Gro ♂ (dashed grey line, full circles) Gro × Gro (black line, triangles), Gro ♀ × Ad ♂ (grey line, full triangles). **Table S1.** Results of binomial tests of the number of mated and not mated individuals for no-choice experiments. **Table S2.** Results of a Mann–Whitney *U*-test between the isolation indexes of each female foundation at each generation assayed, a three-way ANCOVA for the isolation index in female-choice assays across generations and an ANOVA for the isolation index of each female population across generations for the female-choice experiments.
