# Supplementary material for: Evolution of mating behavior between two populations adapting to common environmental conditions
Source: Ecol Evol. 2015 Mar 18;5(8):1609–17. doi: 10.1002/ece3.1454 (PMC4409410; doi:10.1002/ece3.1454)
Supplement: Supplementary file 1 [file ece30005-1609-sd1.docx]

**Appendix to Bárbaro et al. “Evolution of mating behaviour between two populations adapting to common environmental conditions”.**

**Figure S1.** Evolutionary trajectories for percentage of matings in no-choice experiments for Ad x Ad (dashed black line, circles), Ad ♀ x Gro ♂ (dashed grey line, full circles) Gro x Gro (black line, triangles), Gro ♀ x Ad ♂ (grey line, full triangles). Data points show mean values for each block.


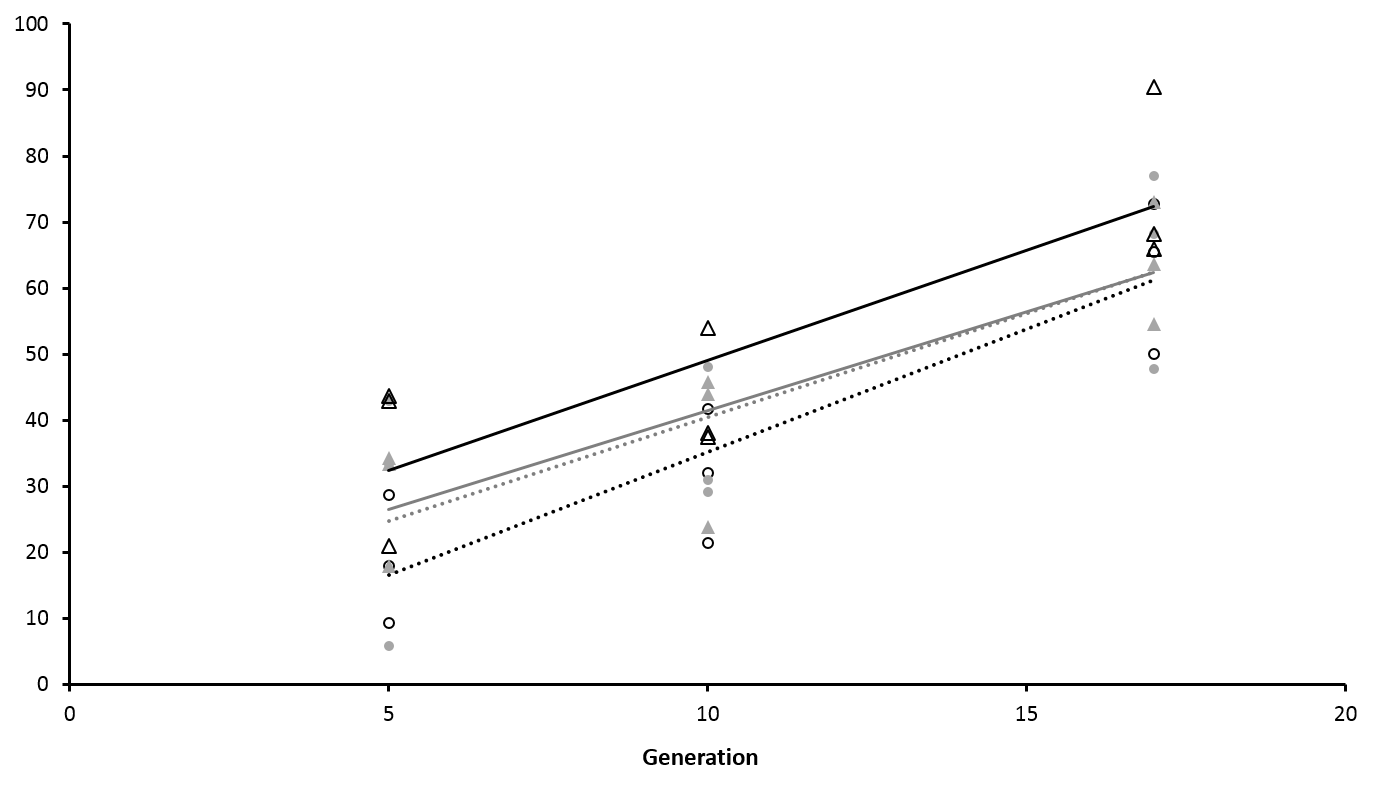


**Table S1.** Results of binomial tests of the number of mated and not mated individuals for no-choice experiments. Data is presented for homogamic crosses at generation 5; and for all crosses at each assayed generation and across generations. Statistically significant factors (*P* < 0.05) are marked in bold.

| **Generation** | **Source** | **Z** | ***P*** |
| --- | --- | --- | --- |
| Homogamic Crosses - 5 | Foundation | 2.930 | **0.0034** |
| 5 | Male Population | 1.710 | 0.0874 |
|  | Female Population | 2.385 | **0.0171** |
| 10 | Male Population | 1.745 | 0.0809 |
|  | Female Population | 1.553 | 0.1204 |
| 17 | Male Population | 1.901 | 0.0574 |
|  | Female Population | 1.901 | 0.0574 |
| Across Generations | Male Population | 3.045 | **0.0023** |
|  | Female Population | 3.276 | **0.0011** |
|  | Generation | 12.045 | **<0.0001** |

**Table S2.** Results of a Mann-Whitney U Test between the isolation indexes of each female foundation at each generation assayed, a three-way ANCOVA for the isolation index in female-choice assays across generations and an ANOVA for the isolation index of each female population across generations for the female-choice experiments. Statistically significant factors (*P* < 0.05) are marked in bold.

| Mann-Whitney U Test | **Generation** | ***Z*** | | ***P*** | |
| --- | --- | --- | --- | --- | --- |
|  | **5** | -1.9640 | | **0.0495** | |
|  | **10** | -1.0911 | | 0.2752 | |
|  | **17** | -1.5275 | | 0.1266 | |
| **ANCOVA across generations** | **Source** | **df** | **MS** | **F** | ***P*** |
|  | Generation | 1 | 0.0059 | 0.1118 | 0.7431 |
|  | Foundation | 1 | 0.3446 | 6.5372 | **0.0228** |
|  | Generation x Foundation | 1 | 0.0397 | 0.7533 | 0.4001 |
|  | Error | 14 | 0.0527 |  |  |
| **ANOVA for each foundation** | **Foundation** | **Source** | | ***P*** | |
|  | Ad | Generation | | 0.4478 | |
|  | Gro | Generation | | 0.6999 | |
